# Redefining atherosclerotic cardiovascular disease patients with no standard modifiable risk factors “SMuRF-less”: six rather than four risk factors. Analysis from the Jordan SMuRF-less Study

**DOI:** 10.3389/fcvm.2026.1785553

**Published:** 2026-08-06

**Authors:** Ayman Hammoudeh, Mo’men Aldalal’ah, Fakhri Al-Malkawi, Enad Haddad, Aseel Rizik, Omar Shadeed, Samia Sulaiman, Mayar Abughosh, Lamia Marouf, Qossay Abu Khalil, Zaid Shbita, Ahmed Alhaj, Jamal M. Hammoudeh, Mohammad Araidah

**Affiliations:** 1 Department of Cardiology, Istishari Hospital, Amman, Jordan; 2 Department of Internal Medicine, Princess Basma Hospital, Ministry of Health, Irbid, Jordan; 3 Department of Internal Medicine, Jefferson Abington Hospital, Abington, PA, United States; 4 Department of Medical Education, University of Jordan School of Medicine, Amman, Jordan; 5 Department of Medical Education, Arab American University School of Medicine, Jenin, Palestinian Territory; 6 Department of Medical Education, University of Szeged Albert Szent Gyorgyi Medical School, Szeged, Hungary; 7 Department of Internal Medicine, Istishari Hospital, Amman, Jordan

**Keywords:** atherosclerotic cardiovascular disease, Middle Eastern cohorts, obesity, physical inactivity, SMuRF-less patients, standard modifiable risk factors

## Abstract

**Background:**

Atherosclerotic cardiovascular disease (ASCVD) is the leading cause of death in Middle East. Some patients sustain events in the absence of the four standard modifiable risk factors (SMuRFs): hypertension, type 2 diabetes, dyslipidemia and cigarette smoking (SMuRF-less patients). Two other modifiable RFs, obesity and physical inactivity, although highly prevalent, are not included in SMuRF-less definition.

**Aim:**

To study the prevalence and clinical profiles of SMuRF-less patients in a large Middle Eastern ASCVD cohort using an expanded definition includes obesity and physical inactivity vs. the traditional four RFs definition.

**Methods:**

We analysed data from the Jordan SMuRF-less study, to compare between expanded and traditional definitions and SMuRF-less vs. SMuRFs groups.

**Results:**

An initial analysis that included 5,540 ASCVD patients [mean age 57.5 ± 11.6 years, and 1,333 (24.1%) were women] showed that 214 (3.9%) patients were SMuRF-less with none of the four RFs. A subgroup of 1,121 patients had available data on their body mass index and physical activity. Of this subgroup, only 11 (1.0%) patients were SMuRF-less with none of the six RFs were present. The 6-RF SMuRF-less patients were younger than those with ≥ 1 SMuRF. These patients also had higher median high-density lipoprotein cholesterol levels and lower median triglycerides serum levels compared with those who have 4–6 RFs.

**Conclusions:**

Including obesity and physical inactivity to the original four RFs lowered the prevalence of “SMuRF-less” by 74.4%. ASCVD prevention strategies need to give more attention to lifestyle-related parameters to avoid misclassifying any modifiable risk factor.

**Clinical Trial Registration**: ClinicalTrials.gov, identifier NCT06199869.

## Introduction

Atherosclerotic cardiovascular disease (ASCVD) is the leading cause of death worldwide, and more than 80% of the global burden of this disease occurs in low-income and middle-income countries, including the Middle East (1, 2). The majority of ASCVD risk is attributable to several key modifiable risk factors (RFs), including four of the most common and highly prevalent RFs; hypertension, type 2 diabetes (T2D), dyslipidemia and cigarette smoking (3). This suggests that global and national strategies to control ASCVD can be significantly enhanced by screening for and managing these RFs across all age groups and genders in different regions of the world (3, 4).

The term “SMuRF-less” (absence of standard modifiable RFs) was first coined by Figtree et al. in the SWEDEHEART Project and refers to patients who suffered myocardial infarction (MI) in the absence of these four SMuRFs (5). Following the publication of the SWEDHEART study, many publications from different regions around the world addressed the prevalence, clinical profiles and outcomes of “SMuRF-less” patients admitted with MI, and they all adopted the same definition of “SMuRF-less” as that defined in the original study (6–8). The list of established RFs for ASCVD is large and continues to expand. These factors include physical inactivity, obesity, autoimmune diseases, genetic predisposition, family history of premature ASCVD, female reproductive age-specific factors, social determinants of health, environmental factors, and psychological stresses (9). Despite this large list of RFs, the definition of “SMuRF-less” continues to include the original four RFs only. A recent study, however, suggested that 60% of incident ASCVD cases were attributable to five SMuRFs, including obesity in addition to the four traditional RFs (10).

The prevalence of ASCVD SMuRF-less patients varies widely across published studies. A meta-analysis of 15 studies involving more than 1.2 million patients with acute coronary syndrome (ACS) revealed that 11.6% were SMuRF-less (11). Other studies reported prevalence rates of SMuRF-less patients as low as 3.6% (7) and as high as 23% of ASCVD patients (12). The only study of SMuRF-less individuals from the Middle East reported a prevalence rate of 3.9% of SMuRF-less patients among 5,540 ASCVD patients (13). The intriguing issue in the definition of SMuRF-less patients is the exclusion of two important standard modifiable RFs and potential drivers of ASCVD, namely, obesity and physical inactivity. These two RFs are highly prevalent at the global level and contribute significantly to the pathogenesis of ASCVD (14). Mounting evidence has shown that efficient intervention for these two factors is an important pillar in planning global and regional strategies for reducing cardiovascular morbidity and mortality and for improving the quality of life of these individuals (10, 14, 15).

There seem to be several explanations for the exclusion of obesity and physical inactivity from the original “SMuRF-less” definition. Some investigators consider these two RFs as risk-enhancing factors rather than factors with independent and causal relationships with ASCVD, and the increased risk of ASCVD related to obesity and physical inactivity is thought to be related to the coexistence of the standard four traditional RFs (14, 16).

In communities with a high prevalence of one or more of the four RFs, the prevalence of SMuRF-less patients is expected to be low. This is likely to be even lower if obesity and physical inactivity are accounted for in the definition of SMuRF-less. Narrowing the circle of ASCVD patients who lack all six RFs will enhance and prioritize strategies for these communities to curb ASCVD by focusing on screening and early intervention for these highly prevalent six, rather than only four RFs.

In this study, we used data from the Jordan SMuRF-less study to propose a redefinition of the current definition of “SMuRF-less” among Middle Eastern cohorts by including obesity and physical inactivity in addition to the original four modifiable RFs. We hypothesize that there will be a significant reduction in the prevalence of SMuRF-less patients with the new proposed definition.

## Methodology

The current analysis evaluated the prevalence and clinical characteristics of SMuRF-less patients who lacked all six modifiable RFs by utilizing data from the Jordan SMuRF-less Study (13). Three groups of ASCVD patients were contrasted: the SMuRF-less group, those with 1–3 SMuRFs, and those with 4–6 SMuRFs. Patients with ASCVD included those with coronary artery disease (CAD), stroke, carotid artery disease and peripheral arterial disease. CAD patients included those with acute coronary syndrome (ACS); i.e., ST-segment elevation MI (STEMI) and non-ST-segment-elevation ACS), chronic coronary syndrome and CAD diagnosed by coronary computed tomography angiography (CCTA).

### Definitions of SMuRFs

HTN diagnosis was based on a previous diagnosis by a physician, the use of antihypertensive medications, or elevated systolic blood pressure ≥140 mm Hg and/or diastolic blood pressure ≥90 mm Hg on entry into the study. T2D was defined as a previous diagnosis, the use of glucose-lowering medications, or a glycated haemoglobin serum level >6.5%. Dyslipidaemia was inferred by a prior diagnosis or the use of lipid-lowering medications. Current cigarette smoking was defined as regular smoking within the past year before study entry. Obesity was defined on the basis of a body mass index (BMI) ≥30 kg/m^2^. Physical inactivity was defined as the absence of regular physical activity (defined as walking for at least 30 min, three times per week).

The study is registered with ClinicalTrials.gov (NCT06199869), was performed in accordance with the Declaration of Helsinki and received Institutional Review Board approval from the participating institutions. Patients provided written informed consent.

### Statistical analysis

Data were analysed via IBM SPSS Statistics version 26. Descriptive statistics were performed using medians and interquartile ranges (IQRs) to describe the continuous variables, and proportions were used to describe the categorical variables. The Kruskal‒Wallis test was used to compare medians, and the chi-square test and Fisher's exact test were used depending on an expected cell count >5 or <5, respectively, to compare percentages of the variables in the three groups of patients according to the number of SMuRFs. A forest plot was used to display the odds ratios and 95% confidence intervals of the use of the pharmacological medications across the three groups of patients. Binary logistic regression analysis was conducted to determine the factors and drugs associated with better one-year survival in the whole cohort, and a forest plot was used to display the odds ratios and 95% confidence intervals of the factors that determine survival with drug distribution. A *p* value of less than 0.05 was considered statistically significant.

## Results

**Figure 1:**
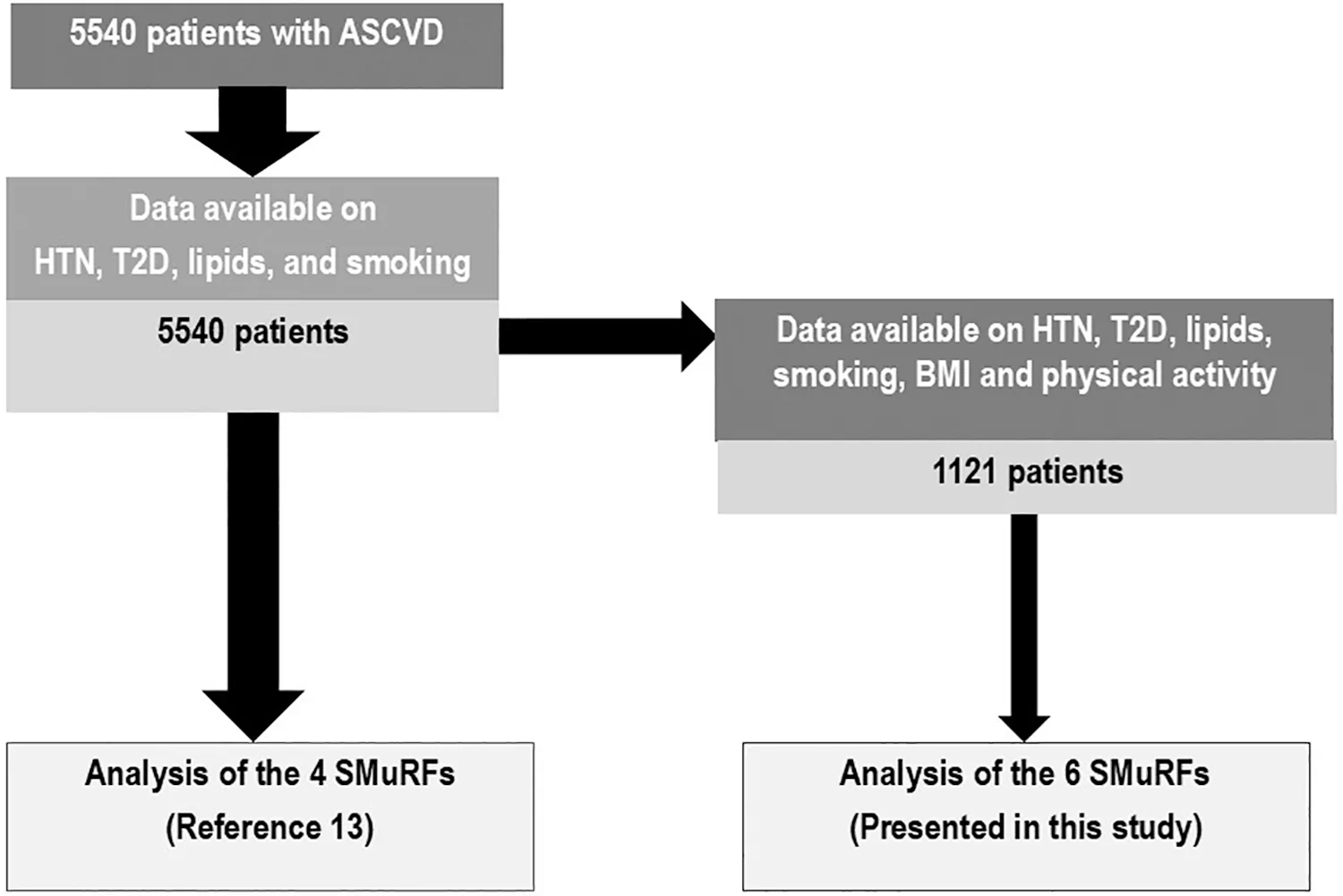
The workflow for the subgroup analysisASCVD: atherosclerotic cardiovascular diseases; BMI, body mass index; HTN, hypertension; SMuRFs, standard modifiable risk factors; T2D, type 2 diabetes.

**Figure 2:**
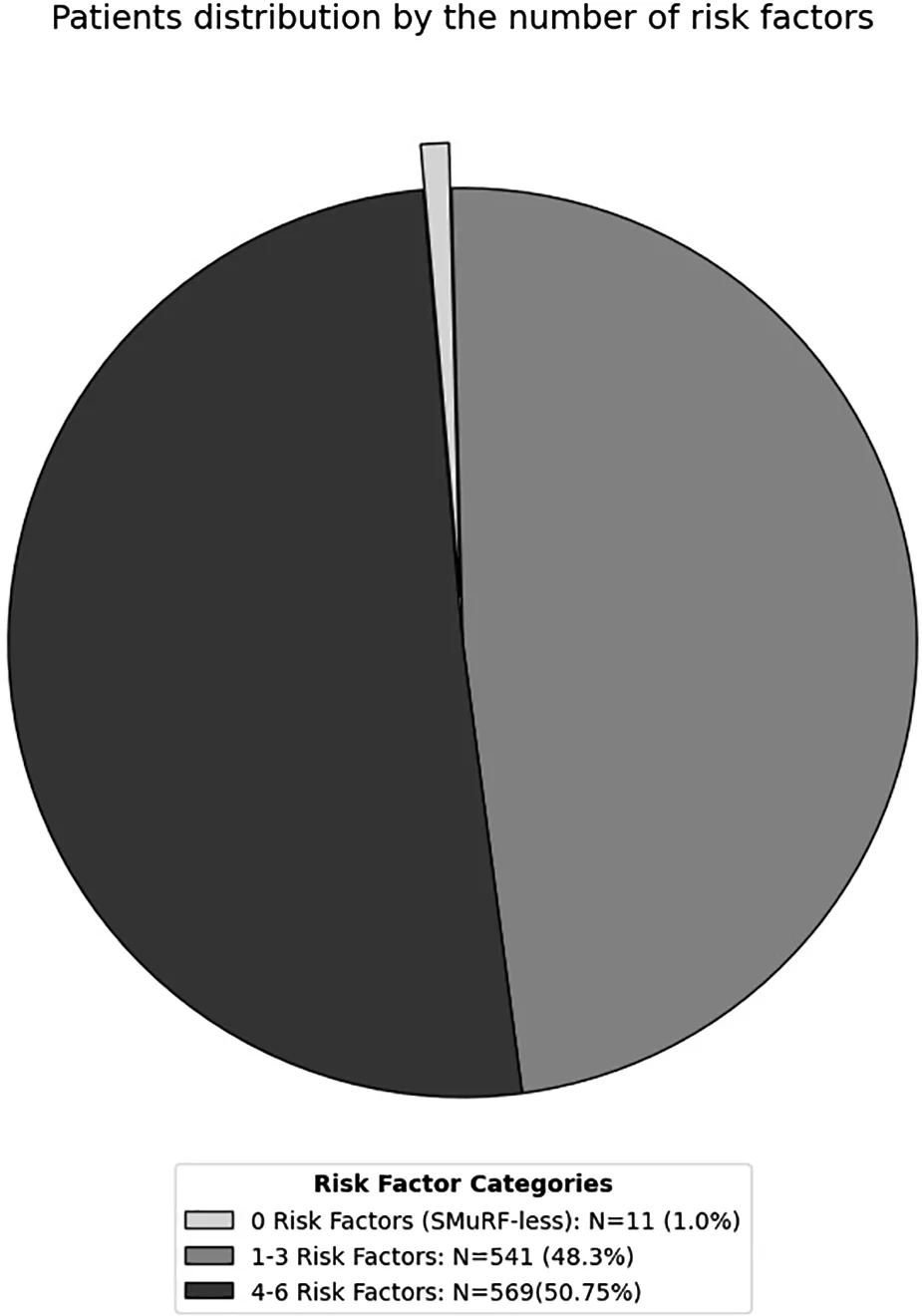
Patients’ distribution by number of risk factors. SMuRFs, standard modifiable risk factors.

The study flowchart is depicted in Figure 1. Among the initial 5,540 patients with ASCVD [mean age 57.5 ± 11.6 years and 1,333 (24.1%) women] who were included in the original study of the four SMuRFs, 3,197 (57.7%) patients had HTN, 2,840 (51.3%) patients had T2D, 4,053 (73.2%) patients had dyslipidaemia, 2,350 (42.4%) patients were cigarette smokers and 214 (3.9%) patients were SMuRF-less (13). BMI measurements were available for 3,600 patients. Among those, physical activity data were available for 1,121 patients whose data were analysed in the current study. There were no significant differences in the baseline characteristics between the 5,540 and 1,120 cohorts. Among the cohort of 1,121 patients, 44 (3.9%) patients were 4-RF SMuRF-less and only 11 (1.0%) were 6-RF SMuRF-less. Thus, including obesity and physical inactivity resulted in a decrease of the proportion of SMuRF-less group by 74.4% (from 3.9% to 1.0%). There were no significant difference in the prevalence of other risk factors between the SMuRF-less patients according to the two definitions, including family history of premature cardiovascular disease (35.4% vs. 35.3%; *p* = 0.949, the presence of heart failure (18.2% vs. 17.7%; *p* = 0.968), chronic kidney disease (14.3% vs. 0.0%, *p* = 0.122) and obstructive sleep apnea (0.0% vs. 5.9%; *p* = 0.411). Figure 2 depicts the distribution of the 1,121 patients by the number of RFs and Table 1 shows the demographic data, clinical profiles of these patients according to the number of RFs. In addition to the 11 SMuRF-less patients, there were 541 (48.3%) with 1–3 RFs and 569 (50.7%) with 4–6 RFs. SMuRF-less patients were younger and had lower prevalence of heart failure, metabolic syndrome and obstructive sleep apnea. The median serum level of high-density lipoprotein cholesterol (HDL-C) was higher, and of triglycerides was lower in the SMuRF-less group in comparison with the other study groups.

**Table 1:** Demographic, clinical and laboratory profiles of three groups of patients according to the number of risk factors.

Variable	No SMuRFs (*n* = 11)	1–3 SMuRFs (*n* = 541)	4–6 SMuRFs (*n* = 569)	Total (*N* = 1,121)	*p*-value
Median Age (IQR)	50 (49–66)	55 (47–67)	59 (50–68)	57 (49–67)	0.0025
Age group					0.005
18–45	2 (18.18%)	118 (21.81%)	74 (13.01%)	194 (17.31%)	
46–65	6 (54.55%)	273 (50.46%)	323 (56.77%)	602 (53.70%)	
>65	3 (27.27%)	150 (27.73%)	172 (30.23%)	325 (28.99%)	
Gender					0.874
Female	6 (54.55%)	307 (56.75%)	331 (58.17%)	644 (57.45%)	
Male	5 (45.45%)	234 (43.25%)	238 (41.83%)	477 (42.55%)	
BMI ≥30	0 (0.00%)	122 (22.55%)	347 (60.98%)	469 (41.84%)	<0.0001
BMI <30	11 (100.00%)	419 (77.45%)	222 (39.02%)	652 (58.16%)	<0.0001
Median BMI (IQR)	29 (25.6–32.6)	25.7 (22.89–27.8)	27.3 (24.52–29.7)	31.22 (27.5–34.6)	<0.0001
ASCVD					
PAD	1 (9.09%)	5 (0.92%)	8 (1.41%)	14 (1.25%)	0.048
CAD	10 (90.91%)	489 (90.39%)	529 (92.97%)	1,028 (91.70%)	0.295
CVA	0 (0.00%)	47 (8.69%)	32 (5.62%)	79 (7.05%)	0.09
SMuRFs					
HTN	0 (0.00%)	221 (40.85%)	502 (88.22%)	723 (64.50%)	<0.0001
Dyslipidemia	0 (0.00%)	357 (65.99%)	529 (92.97%)	886 (79.04%)	<0.0001
Cigarette smoking	0 (0.00%)	180 (33.27%)	288 (50.62%)	468 (41.75%)	<0.0001
T2D	0 (0.00%)	124 (22.92%)	421 (73.99%)	545 (48.62%)	<0.0001
BMI ≥30	0 (0.00%)	122 (22.55%)	347 (60.98%)	469 (41.84%)	<0.0001
Physical inactivity	0 (0%)	284 (52.5%)	491 (86.3%)	775 (69.1%)	<0.0001
Family history of premature CVD	4 (36.36%)	212 (39.19%)	251 (44.11%)	467 (41.66%)	0.235
Comorbidities					
Heart failure	2 (18.18%)	100 (18.48%)	183 (32.16%)	285 (25.42%)	<0.0001
CKD	1 (14.29%)	33 (8.25%)	90 (18.56%)	124 (13.90%)	<0.0001
Depression	1 (16.7%)	22 (9.7%)	18 (10.5%)	41 (10.2%)	0.836
Metabolic syndrome	0 (0%)	129 (26.27%)	305 (59.69%)	434 (42.84)	<0.0001
OSA	0 (0%)	39 (7.9%)	112 (21.7%)	151 (14.8%)	<0.0001
Socioeconomic factors					
Higher education	3 (27.3%)	197 (38.4%)	144 (27.0%)	344 (32.5%)	<0.0001
Urban residence	11 (100%)	401 (77.6%)	417 (77.7%)	829 (77.8%)	0.205
Health insurance	5 (45.5%)	386 (74.8%)	435 (80.9%)	826 (77.6%)	0.002
Novel risk factors (women 18–50 years)					
Preterm delivery	1 (20.0%)	40 (26.9%)	24 (27.0%)	65 (26.8%)	0.942
Hypertensive disease of pregnancy	1 (20.0%)	43 (28.9%)	24 (27.0%)	68 (28.0%)	0.878
Gestational diabetes	1 (20.0%)	22 (14.8%)	17 (19.1%)	40 (16.5%)	0.668
Weight gain after pregnancy	1 (20.0%)	25 (16.8%)	14 (15.7%)	40 (16.5%)	0.956
Premature menopause	1 (20.0%)	16 (10.7%)	10 (11.1%)	27 (11.1%)	0.81
Radiation for breast cancer	0 (0%)	1 (0.7%)	1 (1.1%)	2 (0.8%)	0.916
Autoimmune disease	0 (0%)	14 (7.2%)	5 (3.7%)	19 (5.7%)	0.337
PCOS	0 (0%)	11 (7.3%)	6 (6.7%)	17 (6.9%)	0.811
Labs					
Total cholesterol (median, IQR)	140.5 (137–167)	164 (139–205)	161 (134–204)	163 (137–204)	0.152
LDL-C (median, IQR)	79 (69–86)	97 (74–128)	93 (67–129)	95 (70–128)	0.081
LDL-C ≤ 55	0 (0%)	37 (8.9%)	69 (14.4%)	106 (11.8%)	0.027
Triglycerides (median, IQR)	134 (116–158)	133 (99–177)	161 (114–241)	148 (106–210)	<0.0001
HDL-C (median, IQR)	44 (38–49)	43 (35–52)	39 (32–46)	40 (34–49)	<0.0001
High HDL-C*	3 (50.0%)	186 (48.7%)	138 (31.1%)	327 (39.3%)	<0.0001

SMuRFs, standard modifiable risk factors; IQR, interquartile range; BMI, body mass index; ASCVD, atherosclerotic cardiovascular diseases; PAD, peripheral arterial diseases; CAD, coronary artery diseases; CVA, cerebrovascular accident; HTN, hypertension; T2D, type 2 diabetes; CVD, cardiovascular disease; CKD, chronic kidney disease; OSA, obstructive sleep apnea; LDL-C, Low-Density Lipoprotein cholesterol; HDL-C, high-density lipoprotein cholesterol; PCOS, polycystic ovary syndrome; OSA, obstructive sleep apnea.

* High HDL-C was defined as >40 mg/dL in males and >50 mg/dL in females.

The utilization of major secondary cardiovascular prevention medications in the three groups of patients is shown in Table 2. Overall, oral antiplatelet agents, statins and beta blockers were prescribed for the majority (>70%) of patients. SMuRF-less patients were less likely to be prescribed dual antiplatelet agents, statins, beta-blockers and renin-angiotensin-aldosterone blockers.

**Table 2:** Utilization of major secondary cardiovascular prevention medications.

Medications	No SMuRFs (*n* = 11)	1–3 SMuRFs (*n* = 541)	4–6 SMuRFs (*n* = 569)	Total (*N* = 1,121)	*p*-value
Aspirin	9 (81.8%)	410 (75.8%)	473 (83.1%)	892 (79.6%)	0.010
Clopidogrel	4 (36.4%)	261 (48.2%)	292 (51.3%)	557 (49.7%)	0.399
Other P2Y12 inhibitors	0 (0%)	31 (5.7%)	41 (7.2%)	72 (6.4%)	0.413
DAPs	3 (27.3%)	229 (42.3%)	281 (49.4%)	513 (45.8%)	0.029
Use of any antiplatelet agent	10 (90.9%)	473 (87.4%)	526 (92.4%)	1,009 (90.0%)	0.021
Statins	9 (81.8%)	454 (83.9%)	529 (93.0%)	992 (88.5%)	<0.0001
RAASi	4 (36.4%)	226 (41.8%)	345 (60.6%)	575 (51.3%)	<0.0001
*β*-blocker	5 (45.5%)	367 (67.8%)	462 (81.2%)	834 (74.4%)	<0.0001
OHA	1 (9.1%)	109 (20.2%)	291 (51.1%)	401 (35.8%)	<0.0001
Insulin	0 (0%)	34 (6.3%)	141 (24.8%)	175 (15.6%)	<0.0001

OHA, oral hypoglycaemic agent; RAASi, renin-angiotensin-aldosterone inhibitor; DAP, dual anti-platelet; SMuRFs, standard modifiable risk factors.

## Discussion

This is the one of the earliest studies to challenge and propose expanding the current definition of the “SMuRF-less” term in the Middle Eastern population. The often overlooked group of ASCVD patients without SMuRFs has received increased attention in the last decade in terms of addressing the prevalence, clinical profiles, pathogenesis, and prognosis (4–8). An important drawback of the current definition of “SMuRF-less” is the exclusion of two important and common modifiable RFs, particularly in the Middle Eastern population: obesity and physical inactivity. In this study, we evaluated the prevalence of SMuRF-less patients among a large Middle Eastern cohort with ASCVD on the basis of six, rather than four, modifiable RFs. The major findings of the study are as follows: (a) obesity and physical inactivity were highly prevalent in the studied ASCVD population; (b) the prevalence of SMuRF-less patients dropped dramatically by 74.4% (from 3.9% to 1.0%) when the new proposed definition included six RFs; and (c) the SMuRF-less patients were younger and had more favourable clinical profiles than those who had at least one RF.

The landmark INTERHEART study evaluated the effect of potentially modifiable RFs associated with MI in 52 countries and indicated that obesity and physical inactivity, among eight other standard RFs and lifestyle patterns, accounted for most of the risk of MI worldwide in both sexes and in all regions of the world (3). This finding suggests that approaches to prevent ASCVD and most premature cases of MI can be based on similar principles worldwide by screening for and controlling these RFs (17). The same study showed that obesity risk can be tracked by different parameters, either BMI or Waist-Hip Ratio (WHR). However, WHR is better in cardiovascular risk prediction (18).

Despite the high prevalence of cardiovascular RFs on a global level, including low- and middle-income countries, there is still a group of ASCVD patients with variable prevalence rates who lack the four standard modifiable RFs. The marked differences in the proportion of SMuRF-less patients across the globe might be explained by differences in risk factor definitions and screenings, genetic predispositions, and lifestyle factors such as smoking and physical activity (19). The lower the proportion of SMuRF-less patients in a community, the more focus is needed to be directed at controlling the common modifiable RFs, rather than genetic and rare RFs, in curbing the ASCVD pandemic.

Obesity was not included in the original SMuRF definition by the researchers who initially coined the term, but was termed a “potential modifiable factor” (5, 20), possibly because of the mixed evidence for its direct link to ASCVD. The increased risk of all-cause mortality in individuals with obesity was believed to be dependent on the coexistence of other RFs in these individuals (15, 21). Furthermore, adiposity was not considered to be among the main drivers of advanced atherosclerosis (22). Recently, a growing evidence has demonstrated a more direct causal relationship between obesity and ASCVD (23). Only recently, a few studies suggested considering a five-RF SMuRF-less definition by adding obesity (10, 24). Furthermore, new treatment options for obesity, including bariatric surgery, have been shown to be associated with positive cardiovascular outcomes (17, 20, 25). The causal relationship between adipose tissue in obesity and ASCVD involves multiple pathogenic pathways, including direct effects on the arterial endothelium and the myocardium via the increased expression of proinflammatory and proatherogenic cytokines and adipokines, such as interleukin-10, and obesity-associated epicardial adipose tissue, which contributes to the pathogenesis of coronary atherosclerosis (26, 27). Furthermore, obesity-related insulin resistance and beta-cell dysfunction are associated with the most common RFs, such as dyslipidaemia, T2D, hypertension, and physical inactivity, thus exacerbating cardiovascular risk in individuals with obesity (27). A study analysing the National Inpatient Sample (NIS) database concluded that SMuRF-less patients were significantly less likely to have obesity than SMuRF patients (13.7% vs. 28.0%, respectively) (28).

Globally, 1 in 4 adults is not sufficiently active (29). Sedentary behaviour and physical inactivity are modifiable risk factors for ASCVD, including subclinical atherosclerosis, ACS, stroke and heart failure (29). The original publication of “SMuRF-less” patients did not include physical inactivity in the definition, but recommended documenting a detailed history of physical activity for all ASCVD patients and for individuals who plan to be engaged in moderate or vigorous intensity aerobic physical activity (20). Recently, several studies have demonstrated that physical inactivity is a major modifiable RF for ASCVD across different geographic regions, sexes, and obesity statuses (30, 31). Another study concluded that physical inactivity is associated with a 24% greater risk of coronary heart disease and a 16% greater risk of stroke (31). Conversely, regular exercise exerts positive direct structural and functional benefits on the endothelium, myocardium, coagulation haemostasis, abdominal adiposity, insulin sensitivity, and glycaemic, lipoprotein and blood pressure control (32, 33).

Studies comparing the survival of SMuRF-less patients with that of patients with RFs have yielded conflicting results. A better one-year survival of SMuRF-less patients was reported by few studies (6, 13). Other studies reported equal or worse survival of these patients compared with those who have RFs (5, 7, 8, 11). Such discrepancies in survival are attributed to variations in the age of the patients, geographic area, management strategies, number of RFs, and coexisting comorbid diseases. The number of deaths in the current study was very small, and hence no variable in the multivariate regression analysis was found to be a determining factor for better survival in the SMuRF-less group.

This study has a few limitations that are worth discussing. The findings might have been susceptible to unmeasured confounding and selection bias, as is the case in most clinical registries. Defining “SMuRF-less” is subject to misclassification due to missing RF data at study entry, due to self-reporting, or due to transient blood pressure or serum glucose elevations in patients with ACS. Definitions of obesity and physical inactivity vary among studies, but in this study, we chose the most common and practical definitions. However, the definition of physical inactivity used a relatively simplified definition, the absence of regular walking for at least 30 min, three times per week. It is possible that it led to an underestimate of the burden of physical inactivity in the cohort. However, the data were collected via self-report methodology, which may have introduced recall bias to the results. High utilization of statin medications in ASCVDs as a secondary prevention medication limits the application of dyslipidemia LDL-C classification criteria, LDL-C ≥55. To avoid the effect of statin medication on LDL-C then dyslipidaemia classification, the classification in analysis depended on prior diagnosis of dyslipidaemia or lipid lowering medication prescription prior the ASCVDs event. However, sensitivity analysis (Supplementary Table S1) showed no significant difference in the relative decrease in the percentage of SMuRF-Less patients from 4RF definition to 6RF definition with and without LDL-C ≥55 criteria (76.67% and 74.4%; respectively).Generalising the findings of this study to the general population in this region might not be applicable because the study was conducted at tertiary care centres in a single Middle Eastern country. Even in the country of the study, the generalizability of the results is limited by the fact that the participants were recruited from tertiary care centres. Despite these limitations, the major strength of this study is that it is one of the earliest studies to challenge the current definition of SMuRF-less ASCVD patients in populations with high traditional risk factor prevalence, like the Middle Eastern population. We suggest considering obesity and physical inactivity alongside the original four RFs in defining “SMuRF-less”. This suggestion is based on the high prevalence of these two RFs and on the recent evidence that supports a causal role for obesity and physical inactivity across a range of cardiovascular conditions greater than previously appreciated.

In conclusion, in this Middle Eastern study of a large cohort with ASCVD, the small number of ASCVD patients who were SMuRF-less according to the original definition became even smaller when obesity and physical inactivity were added to the definition of “SMuRF-less”. SMuRF-less cohorts were reduced by 74.4% by using the six RFs definition. This suggests that many of these patients at least one more important modifiable risk factor, either obesity or physical inactivity. While acknowledging the importance of improving the visibility of the clinical profiles, pathogenesis and outcome of SMuRF-less individuals (34), we suggest screening for and controlling all six standard modifiable RFs, especially among Middle Eastern populations. This approach may help physicians to avoid misclassifying patients as low risk when they still carry modifiable lifestyle-related risk factors. This may improve epidemiologic characterisation and have a more positive impact on curbing cardiovascular disease, particularly in Middle Eastern populations. Prospective studies with formal discrimination and risk stratification analyses are needed to modify established SMuRF frameworks and provide recommendations for preventive guidelines.

## Data Availability

The raw data supporting the conclusions of this article will be made available by the authors, without undue reservation.
