# Supplementary material for: Redefining atherosclerotic cardiovascular disease patients with no standard modifiable risk factors “SMuRF-less”: six rather than four risk factors. Analysis from the Jordan SMuRF-less Study
Source: Front Cardiovasc Med. 2026 Aug 6;13:1785553. doi: 10.3389/fcvm.2026.1785553 (PMC13489840; doi:10.3389/fcvm.2026.1785553)
Supplement: Supplementary file 1 [file Table1.docx]

**^Supplementary Material^**

**^Table S1: Reclassify patients using different dyslipidemia definitions^**⊥

|  | **4RFs cohort** | **6RFs cohort** | **Relative decrease in the percentage** |
| --- | --- | --- | --- |
| **SMuRF-Less using different dyslipidemia definitions** | | |  |
| LDL-C ≥ 55^1^ | 0.6% | 0.14% | 76.67% |
| Without LDL criterion^2^ | 3.90% | 1.00% | 74.4% |
| LDL-C ≥ 100^3^ | 4.63% | 1.60% | 65.44% |
| LDL ≥ 130^4^ | 8.3% | 2.81% | 66.14% |

^1^ Dyslipidemia definition :(i) Prior diagnosis - captures patients diagnosed before enrollment. (ii) Use of lipid-lowering medications - captures treated patients regardless of current LDL value (iii) LDL-C ≥55 mg/dL

^2^ Dyslipidemia definition :(i) Prior diagnosis - captures patients diagnosed before enrollment (ii) Use of lipid-lowering medications - captures treated patients regardless of current LDL value.

^3^ Dyslipidemia definition :(i) Prior diagnosis - captures patients diagnosed before enrollment (ii) Use of lipid-lowering medications (iii) LDL-C ≥100 mg/dL

SMuRF: Standard Modifiable Risk Factors; LDL-C: Low- Density Lipoprotein cholesterol; RF: Risk Factor.

^4^ Dyslipidemia definition :(i) Prior diagnosis - captures patients diagnosed before enrollment. (ii) Use of lipid-lowering medications - captures treated patients regardless of current LDL value. (iii) LDL-C ≥130 mg/dL

⊥ This re-classification was done on the 1121 cohort, the patients with available data of obesity and physical inactivity.
